# Bile acid transporters and regulatory nuclear receptors in the liver and beyond

**DOI:** 10.1016/j.jhep.2012.08.002

**Published:** 2013-01

**Authors:** Emina Halilbasic, Thierry Claudel, Michael Trauner

**Affiliations:** Hans Popper Laboratory of Molecular Hepatology, Division of Gastroenterology and Hepatology, Department of Internal Medicine III, Medical University of Vienna, Austria

**Keywords:** Bile acids, Cholestasis, Fatty liver disease, Gallstones, Liver regeneration, Liver cancer, 6-ECDCA, 6-ethylchenodeoxycholic acid, AE2, anion exchanger 2, ABCG5/8, cholesterol efflux pump, ATP-binding cassette, subfamily G, member 5/8, BA, bile acid, AMPK, AMP activated protein kinase, BCRP (ABCG2), breast cancer resistance protein, ATP-binding cassette, subfamily G, member 2, BRIC, benign recurrent intrahepatic cholestasis, BSEP (ABCB11), bile salt export pump, CAR (NR1I3), constitutive androstane receptor, EGFR, epidermal growth factor receptor, FGF15/19, fibroblast growth factor 15/19, FXR (NR1H4), farnesoid X receptor/bile acid receptor, GLP-1, glucagon like peptide 1, GR (NR3C1), glucocorticoid receptor, HCC, hepatocellular carcinoma, HNF1α, hepatocyte nuclear factor 1 alpha, HNF4α (NR2A1), hepatocyte nuclear factor 4 alpha, IBABP (FABP6, ILBP), intestinal bile acid-binding protein, fatty acid-binding protein 6, ICP, intrahepatic cholestasis of pregnancy, IL6, interleukin 6, LCA, lithocholic acid, LRH-1 (NR5A2), liver receptor homolog-1, LXRα (NR1H3), liver X receptor alpha, MDR1 (ABCB1), *p*-glycoprotein, ATP-binding cassette, subfamily B, member 1, Mdr2/MDR3 (ABCB4), multidrug resistance protein 2 (rodents)/3 (human), MRP2 (ABCC2), multidrug resistance-associated protein 2, ATP-binding cassette, subfamily C, member 2, MRP3 (ABCC3), multidrug resistance-associated protein 3, ATP-binding cassette, subfamily C, member 3, MRP4 (ABCC4), multidrug resistance-associated protein 4, ATP-binding cassette, subfamily C, member 4, NAFLD, non-alcoholic fatty liver disease, NASH, non-alcoholic steatohepatitis, norUDCA, norursodeoxycholic acid, NR, nuclear receptor, NTCP (SLC10A1), sodium/taurocholate cotransporting polypeptide, solute carrier family 10, member 1, OATP1A2 (SLCO1A2, OATP1, OATP-A, SLC21A3), solute carrier organic anion transporter family, member 1A2, OATP1B1 (SLCO1B1, OATP2, OATP-C, SLC21A6), solute carrier organic anion transporter family, member 1B1, OATP1B3 (SLCO1B3, OATP8, SLC21A8), solute carrier organic anion transporter family, member 1B3, OSTαβ, organic solute transporter alpha/beta, PBC, primary biliary cirrhosis, PFIC, progressive familial intrahepatic cholestasis, PH, partial hepatectomy, PPARα (NR1C1), peroxisome proliferator-activated receptor alpha, PPARγ (NR1C3), peroxisome proliferator-activated receptor gamma, PSC, primary sclerosing cholangitis, PXR (NR1I2), pregnane X receptor, RARα (NR1B1), retinoic acid receptor alpha, RXRα (NR2B1), retinoid X receptor alpha, SHP (NR0B2), short heterodimer partner, SRC2, p160 steroid receptor coactivator, TGR5, G protein-coupled bile acid receptor, TNFα, tumor necrosis factor α, TPN, total parenteral nutrition, UDCA, ursodeoxycholic acid, VDR (NR1I1), vitamin D receptor. Please note that for the convenience of better readability and clarity, abbreviations for transporters and nuclear receptors were capitalized throughout this article when symbols were identical for human and rodents

## Abstract

Bile acid (BA) transporters are critical for maintenance of the enterohepatic BA circulation where BAs exert their multiple physiological functions including stimulation of bile flow, intestinal absorption of lipophilic nutrients, solubilization and excretion of cholesterol, as well as antimicrobial and metabolic effects. Tight regulation of BA transporters via nuclear receptors is necessary to maintain proper BA homeostasis. Hereditary and acquired defects of BA transporters are involved in the pathogenesis of several hepatobiliary disorders including cholestasis, gallstones, fatty liver disease and liver cancer, but also play a role in intestinal and metabolic disorders beyond the liver. Thus, pharmacological modification of BA transporters and their regulatory nuclear receptors opens novel treatment strategies for a wide range of disorders.

## Introduction

**Fig. 1:**
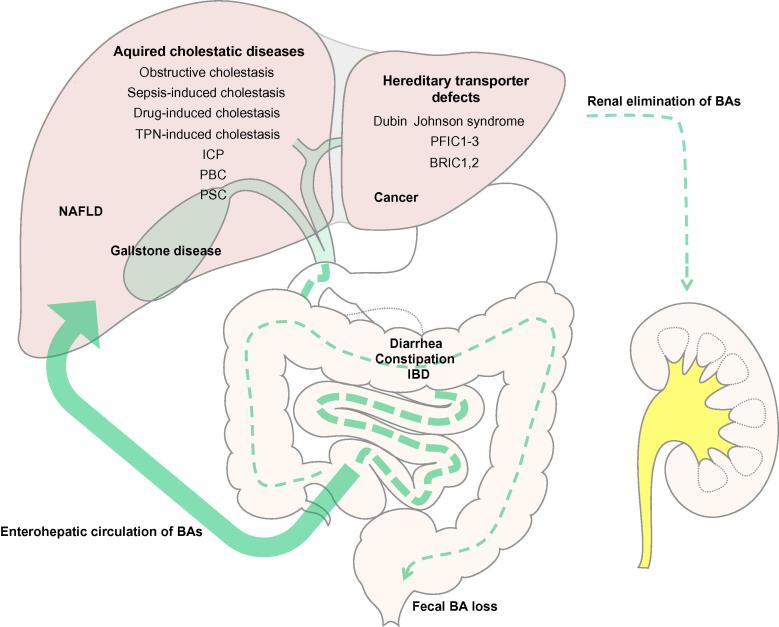
**Overview of diseases linked to disturbances in enterohepatic bile acid circulation**. After their synthesis in hepatocytes, bile acids (BAs) are excreted into the bile and subsequently reabsorbed by enterocytes and, after completing the enterohepatic circulation, by hepatocytes. Efficient reuptake in the ileum preserves 95% of secreted BAs. Disturbances of transport processes within the enterohepatic circulation cause a variety of hepatic and intestinal disorders. Under normal conditions, BAs filtered by the kidney are conserved in the kidney (reabsorption in renal tubules) but can be alternatively excreted when BAs accumulate due to impaired biliary excretion in cholestasis. BAs, bile acids; BRIC, benign recurrent intrahepatic cholestasis; IBD, inflammatory bowel disease; ICP, intrahepatic cholestasis of pregnancy; NAFLD, non-alcoholic fatty liver disease; PBC, primary biliary cholestasis; PFIC; progressive familial intrahepatic cholestasis; PSC, primary sclerosing cholangitis.

To exert their unique physiologic functions bile acids (BAs) undergo enterohepatic circulation requiring active transport processes through the liver and digestive tract [1] (Fig. 1). During this tightly regulated cycle, a minor fraction (less than 3–5%) of secreted BAs escapes intestinal reabsorption via feces and needs to be replaced by *de novo* synthesis [1]. Maintenance of the enterohepatic BA circulation is vital for several liver and gastrointestinal functions including bile flow, solubilization and excretion of cholesterol, clearance of toxic molecules, intestinal absorption of lipophilic nutrients, as well as metabolic and antimicrobial effects [2]. Moreover, the enterohepatic circulation efficiently preserves these precious molecules, since BA synthesis from cholesterol involves 17 energy-consuming enzymatic steps [3]. In the body, BAs are mainly present in their conjugated form, which prevents unrestricted diffusion; therefore, BAs must be transported via energy-driven transport systems across the membranes of cells involved in the enterohepatic circulation [4]. BA transporters have different transport affinities for various BA species, but also for other endogenous and exogenous compounds such as drugs and toxins (Table 1). The expression of genes involved in BA homeostasis is tightly controlled by nuclear receptors (NRs) which sense the intracellular concentrations of BAs; in addition, post-transcriptional mechanisms such as insertion/retrieval of transporters into/from the cell membrane regulate the transport capacity via protein kinase C and mitogen-activated protein kinase activation by BAs [5–8]. Together with transcriptional regulation, such post-transcriptional changes fine-tune transporter expression and activity at the plasma membrane (recently reviewed in [9]). In addition to NRs as intracellular BA sensors, some cells also contain BA receptors at the cell surface including a G-protein coupled receptor (TGR5/M-BAR/GPBAR1) [10] and the epidermal growth factor receptor (EGFR) [11]. Under physiological conditions, these regulatory networks preserve the enterohepatic BA circulation and limit intracellular levels of potentially toxic BAs. Disturbances of this delicate balance may contribute to cholestasis, gallstone disease, malabsorption and intestinal bacterial overgrowth (Fig. 1). By determining the distribution of BAs as signaling molecules with hormonal functions, transporter alterations also play a key role in fatty liver disease, insulin resistance, liver regeneration and cancer (Fig. 1). Modification of transporters and regulatory NRs may be utilized to develop novel therapeutic and preventive pharmacological strategies for these diseases. This review provides a comprehensive summary of the latest advances in understanding the function of hepatobiliary transporters and their key regulatory NRs for BA homeostasis in health and diseases, highlighting the potential clinical and therapeutic implications.

## Hepatocellular bile acid transporters and their regulation by nuclear receptors

### Basolateral uptake systems in the liver

**Fig. 2:**
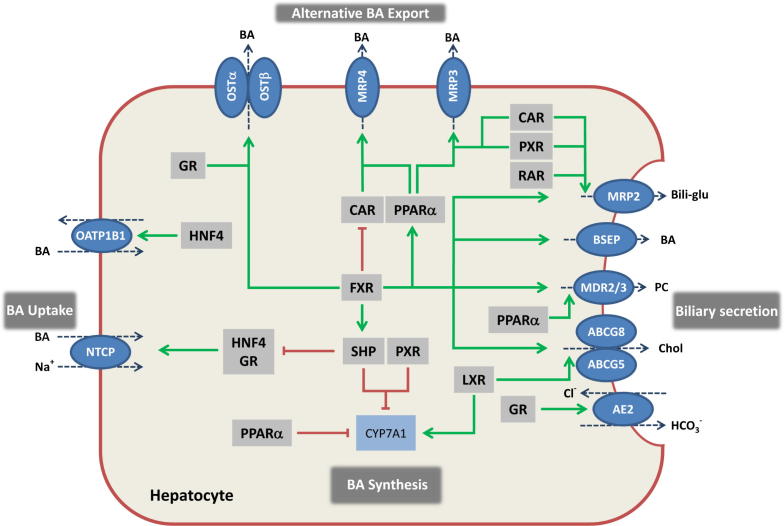
**Transcriptional regulation of hepatocellular bile formation**. Expression of hepatobiliary transporters in hepatocytes determines hepatic bile acid (BA) flux and hepatocellular concentrations of these potentially toxic metabolites. To ensure the balance between synthesis, uptake and excretion, expression of hepatobiliary transporters is tightly regulated by nuclear receptors (NRs). NRs provide a network of negative feedback and positive feed-forward mechanisms, for the control of intracellular concentration of biliary constituents, which are often also ligands for these NRs. BA-activated FXR is a central player in this network, that represses (via interaction with HNF4 in rats and GR in humans) hepatic BA uptake (NTCP) and (via SHP) synthesis (CYP7A1), promotes bile secretion via induction of canalicular transporters (BSEP, MRP2, ABCG5/8, MDR3) and induces BA elimination via alternative export systems at the hepatic basolateral (sinusoidal) membrane (OSTα/β). Several NR pathways converge at the level of CYP7A1 as the rate limiting enzyme in BA synthesis. CAR and PXR facilitate adaptation to increased intracellular BA concentrations by upregulation of alternative hepatic export routes (MRP3 and MRP4) and induction of detoxification enzymes (not shown). Together with RAR, these xenobiotic receptors also regulate the canalicular expression of MRP2. Cholesterol sensor LXR promotes biliary cholesterol excretion via ABCG5/8. Stimulation of AE2 expression by GR stimulates biliary bicarbonate secretion thus reducing bile toxicity. Green arrows indicate stimulatory and red lines suppressive effects on target genes. In addition to these transcriptional mechanisms, post-transcriptional processes (e.g., vesicular targeting of transporters to the membrane, phosphorylation of transport proteins) and modification of the bile through cholangiocytes (e.g., bicarbonate secretion) also play an important role in bile formation (not shown). BAs, bile acids; Bili-glu, bilirubin glucuronide; BSEP, bile salt export pump; CAR, constitutive androstane receptor; CYP7A1, cholesterol-7α-hydroxylase, FXR, farnesoid X receptor; GR, glucocorticoid receptor; HNF4, hepatocyte nuclear factor 4, LXR, liver X receptor; MDR3, multidrug resistance protein 3, phospholipid flippase; MRP2, multidrug resistance-associated protein 2; MRP3, multidrug resistance-associated protein 3; MRP4, multidrug resistance-associated protein 4; NTCP, sodium taurocholate co-transporting polypeptide; OSTα/β, organic solute transporter alpha and beta, PC, phosphatidylcholine; PXR, pregnane X receptor; PPARα, peroxisome proliferator-activated receptor alpha; RAR, retinoic acid receptor; SHP, small heterodimer partner.

BAs return to the liver via portal blood (and to a much lesser extent via the hepatic artery) and are efficiently removed during their first passage through the hepatic sinusoids by hepatocellular BA uptake systems [12], involving a sodium-dependent sodium/taurocholate co-transporting polypeptide (NTCP/SLC10A1) and a family of sodium-independent multispecific organic anion transporters (OATPs/SLCOs) [13,14] (Fig. 2; Table 1).

NTCP accounts for the bulk (about 90%) of BA uptake and was the first cloned BA transporter [13]. Its regulation under physiological and pathological conditions is therefore well understood thus serving as a paradigmatic model to understand transporter regulation. NTCP expression is controlled by BAs, hormones such as estrogen and prolactin, as well as pro-inflammatory cytokines (recently reviewed in [15]). In cholestatic patients [16,17] and animal models of cholestasis induced by biliary obstruction, estrogen or endotoxin, NTCP expression is universally reduced (reviewed in [18]). A key repressive mechanism involves activation of the farnesoid X receptor (FXR) through accumulating BAs, which then induces the small heterodimer partner (SHP) as repressor of hepatic nuclear factor 1 alpha and 4 alpha (HNF-1α and HNF-4α, and also interfering with retinoid X receptor (RXR), retinoic acid receptor (RAR) heterodimers in rats, or the glucocorticoid receptor in humans (recently reviewed in [15]), which are all required for normal NTCP expression (Fig. 2, Table 1).

### Canalicular export systems

At the canalicular membrane, highly specialized canalicular transporters mediate excretion of the individual components of bile such as BAs, phospholipids and cholesterol [4] (Fig. 2, Table 1). The bile salt export pump (BSEP, ABCB11 or sister of *p*-glycoprotein (Spgp)) is the major canalicular BA efflux system [19]. The relevance of BSEP is emphasized by the severe progressive familial cholestatic syndrome (PFIC2) or benign recurrent intrahepatic cholestasis (BRIC2) resulting from *BSEP* mutations (Table 1). Importantly, the functional implications of BSEP deficiency may be underestimated in knockout mice (Table 1) where BA composition is less toxic than in humans, and BAs can be excreted by other canalicular transporters (Table 1). BSEP expression/activity is tightly controlled at transcriptional and post-transcriptional levels. FXR [20] upregulates BSEP expression (recently reviewed in [21]). While BSEP is downregulated by inflammatory injury and estrogen, it is relatively well preserved in obstructive cholestasis, which may help limit intracellular BA accumulation, although a preserved bile flow may cause bile infarcts in biliary obstruction (recently reviewed in [15]).

The canalicular membrane also contains transport systems mediating excretion of biliary phospholipids (MDR3, Mdr2 in rodents, ABCB4) and cholesterol (two half transporters ABCG5/8 which are tightly coupled with BA excretion [22] (Fig. 2, Table 1). Other canalicular transport systems (Fig. 2, Table 1) are less relevant for BA transport. Multidrug resistance-associated protein 2 (MRP2/ABCC2) mainly excretes bilirubin–glucuronides and glutathione conjugates, but also divalent sulfo-conjugated BAs into the bile (Fig. 2, Table 1) (Table 1). Multidrug resistance protein (MDR1, ABCB1) primarily excretes lipophilic cations including diverse drugs and carcinogens [23], while breast cancer resistance protein (BCRP, ABCG2) facilitates the transport of potentially toxic xenobiotics and food-derived carcinogens [24] (Table 1). Both transporters have also been implicated in BA transport when induced under cholestatic conditions, although this is still disputed in humans.

### Alternative basolateral efflux systems in hepatocytes

During hepatocellular BA overload, BAs can also be transported back to the sinusoidal blood to protect the liver and for subsequent elimination via the urine. Usually this step is coordinated with phase I and II detoxification, providing less toxic and higher affinity substrates for the basolateral BA export systems [18]. This alternative basolateral BA export is mediated by the multidrug resistance-associated proteins MRP3 (ABCC3), MRP4 (ABCC4) and the heterodimeric organic solute transporter OSTα/OSTβ. Constitutive androstane receptor (CAR; NR1I3), pregnane X receptor (PXR; NR1I2), vitamin D receptor (VDR, NR1I1) and peroxisome proliferator-activated receptor alpha (PPARα, NR1C1) all increase MRP3 expression in mice, while MRP4 is induced by CAR and PPARα (recently reviewed in [15]) (Fig. 2, Table 1). The heterodimeric transporters OSTα/OSTβ were initially identified as an intestinal BA efflux system in enterocytes (see below), but are also found in the liver; their expression is induced via FXR (reviewed in [25]) (Fig. 2, Table 1).

## Cholangiocytes and bile acid transport

Bile duct epithelial cells (cholangiocytes) are important modifiers of bile formation by promoting bicarbonate excretion and line the bile ducts as drainage system for BAs to the intestine. Side chain modified BAs such as norUDCA with a relative resistance to conjugation, can bypass the enterohepatic circulation by a process termed cholehepatic shunting [26]. This process, together with potential direct effects of norUDCA on cholangiocyte secretion, induces bicarbonate-rich hypercholeresis that may represent a drugable protective mechanism in cholangiopathies [27]. In contrast, conjugated BAs require active transport into cholangiocytes via an apical sodium dependent BA transporter (ASBT), identical to the transport system in the ileum (see below). After uptake, BAs are exported into the adjacent peribiliary capillary plexus via OSTαβ and MRP3, and possibly a truncated version of ASBT (tABST) (recently reviewed in [28]). Upregulation of cholangiocellular BA transport capacity in obstructive cholestasis ([28]), partly by bile duct proliferation, may facilitate the removal of BAs from the stagnant bile. Under physiological conditions, a major role of BA transporters in cholangiocytes could be the regulation of intracellular concentrations of BAs as signaling molecules. Notably, several nuclear receptors such as FXR, RXR, LXR, VDR, PPARδ, and SHP, known to play a key role in the regulation of metabolic processes, are also expressed in cholangiocytes, although their role in bile duct (patho)biology remains to be clarified.

## Intestinal bile acid transporters

Apart from a relatively small proportion of passive uptake in the proximal small intestine and colon, BAs are mainly actively taken up in the terminal ileum via ASBT [29,30]. Notably, enterocytes, cholangiocytes and renal tubular cells share several BA transport systems including ASBT [18]. Unlike rats, human and mouse ASBT is under negative feedback regulation by BAs via FXR and SHP (reviewed in [25]). After uptake, BAs are bound to the cytosolic ileal BA binding protein IBABP (also known as ileal lipid binding protein ILBP and fatty acid binding protein 6, FABP6) and exported into the portal blood via OSTα/OSTβ [25]. The colon is exposed to BAs escaping ileal reabsorption and possesses detoxification and efflux systems (e.g., OSTα/OSTβ) for defense against secondary (unconjugated) BAs formed by the intestinal flora [18].

After uptake into enterocytes, BAs induce FGF15 in mice (a homolog of human FGF 19) which acts in an endocrine fashion to repress the BA synthesis in hepatocytes [31], facilitates gallbladder refilling [32] and in a paracrine manner downregulates ASBT expression [33], altogether leading to reduction of circulating BAs. Although under physiological conditions FGF19 originates mainly from enterocytes, patients with obstructive cholestasis show a profound increase in hepatic FGF19 expression (not observed in rodents) that correlates with elevated serum FGF19 levels [34]. In addition, FGF15/19 may also be actively involved in energy homeostasis since it stimulates hepatic glycogen and protein synthesis, as well as β-oxidation without inducing lipogenesis [35]. Apart from its signal function in the portal axis, FGF19 is also secreted into the bile and could have other signaling functions in exposed cells of the biliary and enteric tract [36]. Secretion of glucagon-like peptide 1 (GLP-1) from enteroendocrine cells is mediated by the plasma membrane BA activated G-protein-coupled receptor TGR5 and represents a link between BA and glucose metabolism, since GLP-1, secreted after food intake, facilitates glucose-induced insulin secretion [37].

## Role of bile acid transporters in cholestasis – pathophysiological and therapeutic considerations

Transporter alterations in cholestasis may be primary/pro-cholestatic (e.g., genetic defects (Table 1), inhibition by drugs, repression by cytokines and oxidative stress), or more often represent secondary/adaptive changes attempting to minimize liver injury [18]. In addition to transcriptional and post-transcriptional transporter changes, impaired canalicular contractility and increased tight junction permeability may also contribute to cholestasis [38].

Pro-inflammatory cytokines and oxidative stress impair hepatobiliary transport function at transcriptional and post-transcriptional levels (as recently reviewed in [39,40]). Such mechanisms are obviously relevant for sepsis/infection-induced cholestasis, but also play a role in cholestatic hepatitis caused by drugs and viruses, autoimmune injury, and during obstructive cholestasis with bacterial overgrowth and translocation. A coordinated hepatic (negative) acute phase response to inflammation results in rapid reduction of bile formation via downregulation of both basolateral BA uptake (NTCP, OATPs) and canalicular efflux systems (MRP2 and - to a lesser degree – BSEP). Repression of key regulatory transcriptional networks (HNF1, RXR/RAR, FXR, CAR and PXR) [15] together with post-transcriptional changes such as impaired targeting and/or retrieval of efflux proteins from the canalicular membrane [41], all contribute to the reduced transporter expression. Reduction of the canalicular bilirubin export pump MRP2 in response to LPS [42] may explain hyperbilirubinemia, a well known poor prognostic sign in sepsis. Despite many clinically relevant cholestatic conditions associated with inflammation, only few studies have addressed this in humans. As such, basolateral (NTCP, OATP1B1) and canalicular transporters (BSEP and MRP2) are reduced in jaundiced patients with severe alcoholic hepatitis [43]; MRP2 expression and localization are also impaired in chronic hepatitis C [44].

Total parenteral nutrition (TPN)-induced cholestasis is accompanied by a reduced bile flow in animal models [45], which may be explained by downregulation of BSEP, MRP2 and Mdr2, following repression of regulatory NRs (FXR, CAR and PXR) [46]. In addition, stigmasterol, a soy-derived lipid, antagonizes FXR activity [47]. Interestingly, the addition of soybean fat emulsion prevents hepatic injury by TPN possibly by stimulating PPARα [48]. Notably, intensive insulin therapy reduces cholestasis and biliary sludge in critically ill patients [49], which may be linked to regulation of FXR expression by glucose [50].

Certain drugs (e.g., bosentan, rifampicin, glibenclamide, cyclosporine A) induce liver injury with cholestasis. Although drug-induced (cholestatic) liver injury in most cases is idiosyncratic (e.g., rifampicin), some of the drugs, such as cyclosporine and bosentan, can cause cholestasis via dose-dependent inhibition of BSEP [51]. Other drugs/metabolites such as estradiol 17β-glucuronide inhibit BSEP only after secretion into the canalicular lumen via MRP2 (trans-inhibition) [51], thus constituting a pivotal mechanism for oral contraceptive-induced cholestasis. Patients with *BSEP* variants may be predisposed to intrahepatic cholestasis of pregnancy and cholestasis induced by oral contraceptives or hormone replacement (Table 1). Moreover, a frequent *BSEP* polymorphism resulting in reduced expression [52], has been associated with increased risk for cholestatic side effects by β-lactam antibiotics, psychotropic drugs and proton pump inhibitors [53] (Table 1).

In chronic forms of cholestatic liver injury (e.g., PBC and PSC) secondary transporter changes, which may help adapt to accumulating BAs in cholestasis, dominate the picture. These transporter alterations are characterized by downregulation of uptake systems (NTCP and OATPs) and upregulation of basolateral bile acid export systems (MRP3, MRP4, OSTα/OSTβ) [16,54–56]. In addition, by NR-mediated increases in hydroxylation (phase I) and conjugation (phase II) enzymes catalyzing a detoxification process producing less toxic, more hydrophilic Bas, accompany these transporter changes [54]. Such changes may also explain the biochemical hallmarks of cholestasis, such as elevations in serum BA levels and conjugated bilirubin, resulting in jaundice. Notably, these adaptive transporter changes are not restricted to hepatocytes, but also occur in cholangiocytes, enterocytes and renal tubular cells [18] to maximize the escape of BAs under cholestatic conditions.

Animal studies suggest that most of these adaptive alterations in hepatobiliary transporter systems during cholestasis are mediated by regulatory NR pathways controlled by FXR, VDR, CAR, and PXR, which are activated in response to accumulating BAs and bilirubin [18,57]. Apparently, these coordinated intrinsic NR-triggered defense mechanisms are incapable of sufficiently rescuing the liver from chronic cholestatic injury with progression towards fibrosis and ultimately cirrhosis. Part of these shortcomings may be attributed to impairment of the NR machinery itself by cholestatic liver injury [54].

Pharmacologic NR agonists can stimulate the defective transporter functions and induce additional detoxification pathways [58]. Ironically, ursodeoxycholic acid (UDCA), the only FDA-approved drug for the treatment of cholestasis, has poor NR affinity [20,59]. Nevertheless, UDCA has a broad spectrum of actions including stimulation of the expression of BA detoxifying enzymes and transporters [60] in addition to beneficial immunomodulatory, anti-apoptotic and cytoprotective properties [61]. The effects of UDCA on hepatobiliary transporters appear to be regulated mainly at post-transcriptional levels such as stimulation of transporter targeting [62], while its transcriptional effects are limited. As such, UDCA is only a weak ligand for GR [59] and FXR [20] and indirectly (after enzymatic modification to LCA by intestinal flora) PXR [63]. These effects result in a coordinated stimulation of canalicular transporter (BSEP and MRP2) and basolateral export pumps (MRP3, MRP4, OSTα/β) as demonstrated in rodents and patients [60,64,65]. UDCA and dexamethasone, both synergistically target GR and induce the expression and activity of the anion (chloride/bicarbonate) exchanger 2 (AE2) in hepatocytes and cholangiocytes through coordinated HNF1α/GR/p300 activation [66]. Notably, AE2 is reduced in early PBC [67] with subsequent impairment of biliary bicarbonate secretion [68,69]. This concept is further corroborated in *AE2* knockout mice with immunologic and some morphologic features of PBC [70]. The combination of UDCA therapy with budesonide may be superior to UDCA monotherapy in PBC patients [71] through such synergistic effects on AE2 expression via GR [66].

Novel synthetic BAs are promising alternatives for treatment of cholestatic disorders. 6-Ethylchenodeoxycholic acid (6-ECDCA, INT747), is a specific FXR ligand and highly promising drug candidate for cholestatic liver diseases [72]. FXR agonists improve cholestasis in experimental animals [73], by stimulating hepatocellular BA efflux via induction of BSEP and basolateral overflow systems (via OSTα/β, while lowering endogenous BA synthesis and hepatic BA uptake. FXR activation also increases the expression of MDR3 [74] which may reduce biliary BA toxicity through increased phospholipid excretion with formation of mixed micelles. Similarly, PPARα induces MDR3 mRNA expression and redistributes the localization of the protein to the canalicular membrane [75] which could contribute to the beneficial effects of fibrates in PBC patients with a suboptimal response to UDCA [76,77]. However, it should be considered that MDR3 is already highly expressed in PBC patients [16] and may not be further increased by bezafibrate [78]. In recent phase II clinical trials in patients with PBC, 6-ECDCA alone or in combination with UDCA had beneficial effects on cholestasis, despite development of pruritus at higher doses [72]. Targeting FXR with a dual FXR/TGR5 agonist, INT767, in the *Mdr2* (human MDR3) knockout mouse cholangiopathy model, uncovered FXR-induced bicarbonate secretion as a key therapeutic mechanism [79].

A side chain shortened modification of UDCA, norUDCA, with relative resistance to amidation, represents a promising treatment for cholangiopathies as revealed in the *Mdr2* (human MDR3) knockout mouse model resembling PSC [80]. norUDCA is anticholestatic, antifibrotic and anti-inflammatory and undergoes cholehepatic shunting, also inducing a bicarbonate-rich and potentially less toxic bile flow [27,81]. Moreover, norUDCA improves cholestatic liver injury via marked upregulation of phase I and phase II detoxification enzymes and the alternative basolateral efflux system, and subsequent increase in renal BA excretion [27,80] and restores deranged hepatic lipid metabolism in these mice [82]. So far, no NR targeted by norUDCA has been identified.

PXR (e.g., rifampicin) and CAR (e.g., phenobarbital and Yin Chin, a traditional Chinese herb) ligands have been used to treat pruritus and/or jaundice, long before their exact molecular mode of action was identified [77,83,84]. Activation of PXR and CAR induces the BA and bilirubin detoxification machinery via phase I and II enzymes and subsequent elimination via alternative hepatocellular efflux pumps in animals models of cholestasis [85] and humans [60].

Collectively, changes in BA transporters constitute a central element in the pathogenesis and clinical manifestation of cholestasis and several clinically effective drugs modulate transporter expression via regulatory NRs, translating into a reduction of the hepatocellular BA burden thus ameliorating cholestasis.

## Gallstone disease

High cholesterol or low BA and phospholipid concentrations, as well as BA and phospholipid species, determine the lithogenicity of bile [86]. Therefore, canalicular transporters for cholesterol (ABCG5/8), BAs (BSEP) and phosphatidylcholine (MDR3/Mdr2 in rodents) and their regulatory NRs are relevant for gallstone formation. Quantitative trait locus analysis in gallstone susceptible mice defined *ABCG5/G8* as candidate genes for gallstone susceptibility [87] and its variants were linked to gallstone disease in Chinese, German, Chilean, Indian and Swedish populations (recently reviewed in [88]). Moreover, the expression of ABCG5/8 and its regulatory NR liver X receptor (LXR) (Fig. 2) was increased and correlated with cholesterol saturation in Chinese non-obese gallstone patients [89]. The role of LXR in gallstone formation is further emphasized by transgenic mice which are susceptible to cholesterol gallstone formation [90]. A possible explanation for the link between the metabolic syndrome and gallstones comes from mice lacking the hepatocellular insulin receptor. Increased gallstone formation in these mice has been attributed to increased biliary cholesterol excretion resulting from upregulation of ABCG5/8 due to induction of the forkhead transcription factor FoxO1 and downregulation of BA synthesis [91].

BRIC 2 patients with mutations of *BSEP* are predisposed to gallstones (Table 1), while its hepatic overexpression in mice increases the risk of cholesterol gallstone [92]. The role of MDR3 is underlined by spontaneous gallstone formation in mice lacking *Mdr2* (MDR3 in humans) [93] and “low phospholipid-associated cholelithiasis” in humans with *MDR3* mutations [94]. However, a large population study did not reveal significant associations between *BSEP* or *MDR3* variants and gallstone disease [95]. FXR (a known inducer of BSEP and MDR3) is litho-protective and mice lacking *FXR* are prone to form gallstones [96]. Conversely, pharmacological activation of FXR counteracts gallstone formation [96]. A distinct *FXR* genetic variant was associated with gallstone disease in male Mexicans, whereas no such links were found in Chilean and German populations [97], emphasizing the complexity of multiple genetic factors determining the gallstone disease.

Intestinal BA transporters may also be involved in determining the BA pool size as key factor of cholesterol homeostasis. As such, downregulation of ASBT [98], as wells as OSTα/β (Table 1), was found in gallstone patients. In addition, *ASBT* polymorphisms represent a risk factor for gallstone disease [99].

## Pathophysiologic and therapeutic significance of intestinal bile acid transport and signaling

Inflammatory cytokines downregulate ASBT via the activator protein 1. This may explain BA malabsorption in patients with ileal inflammation and animal models of ileitis, as well as downregulation of ASBT in patients with Crohn’s disease [100]. Whether this contributes to diarrhea and possibly also BA-induced carcinogenesis in the colon, remains to be identified. Notably, *ASBT* polymorphisms have been associated with an increased risk of colorectal adenoma [101], although no association with colorectal carcinoma was found [102]. Under normal conditions, BA-activated FXR controls bacterial overgrowth and maintains the epithelial barrier integrity by induction of multiple anti-inflammatory genes [103] and pharmacological activation of FXR has beneficial effects in the mouse model of colitis [104]. Moreover, intestinal overexpression of FXR protects the intestine from cholestasis-associated mucosal injury and reduces cholestatic liver injury, the latter by repression of bile acid synthesis via the FXR-FGF15/19-FGRR4-CYP7A1 axis [105].

The interruption of the enterohepatic circulation by BA sequestrants may be used therapeutically to eliminate BAs or other pruritogens in the treatment of cholestatic pruritus. Notably, serum BA levels correlate only poorly with the degree of cholestatic pruritus and a recently identified product of the lysophospholipase autotaxin, lysophosphatidic acid, may be a major mediator of pruritus [106]. Furthermore, BA sequestrants are used to treat BA-induced diarrhea and metabolic disorders such as hyperlipidemia and diabetes. They have been shown to improve the glycemic control in diabetic patients [107], by mechanisms involving secretion of GLP-1 [108]. Long before BA sequestrants were used in the treatment of diabetes, their beneficial effects in hypercholesterolemia and atherosclerosis became apparent [109]. Similarly, ileal bypass surgery also reduced overall mortality from coronary heart disease [110]. The results of these studies can now be explained by decreased FGF19 signaling, and derepression of CYP7A1 increasing the conversion of cholesterol to BAs. Hepatic depletion of cholesterol then increases the sterol regulatory element-binding protein 2, which in turn induces the LDL receptor and lowers the LDL cholesterol. In analogy to BA sequestrants, ASBT inhibitors are attractive candidate compounds to treat hypercholesterolemia and atherosclerosis [111,112], but their use may be limited by diarrhea. Nevertheless, this ‘side effect’ may be used to treat constipation [113], since BAs promote colonic fluid and electrolyte secretion [26,114] and some forms of idiopathic constipation have recently been linked to impaired colonic BA metabolism/signaling [115,116]. Conversely, up to one third of patients with chronic diarrhea may suffer from BA malabsorption [117], which may be successfully treated with BA binding resins [118].

## Bile acid transporters and fatty liver disease

During the last decades, it has become apparent that BAs are not only detergents, but also possess a number of hormonal effects on lipid and glucose metabolism/storage (recently reviewed in [119]). BAs, via FXR and its downstream targets including SHP, control hepatic *de novo* lipogenesis, very low density lipoprotein-triglycerides export and plasma triglyceride turnover. BA-activated FXR is also involved in the regulation of hepatic gluconeogenesis, glycogen synthesis and insulin sensitivity. Postprandially, when serum BA concentration increase, BAs escape from the enterohepatic circulation by spillover and thereby reach tissues such as adipose tissue and skeletal muscle, usually not exposed to significant BA levels during fasting. via TGR5, BAs are able to stimulate GLP-1 secretion in the small intestine, and energy expenditure in brown adipose tissue and skeletal muscle [120].

Since hepatobiliary transporters critically determine hepatic BA flux and concentrations, it is attractive to hypothesize that alterations of BSEP – the rate limiting step for BA efflux – could contribute to the pathogenesis of non-alcoholic fatty liver disease (NAFLD). Indeed, mice overexpressing BSEP have increased biliary lipid secretion and are protected from steatosis when fed an atherogenic diet [121]. In line with this, BSEP over-expression lowered hepatic lipid accumulation, but not inflammation in mice fed with a methionine–choline deficient diet, as model for steatohepatitis [122]. Mice lacking *FXR* with low BSEP expression, spontaneously develop hepatic steatosis and hypertriglyceridemia associated with insulin resistance, emphasizing the role of BA signaling in lipid and glucose metabolism (recently reviewed in [73,120]). Recent studies have reported a positive association between *BSEP* variants and increased serum triglycerides and cholesterol levels [123] and obesity [124] in humans. In contrast to the association between common *BSEP* polymorphisms and cirrhosis in hepatitis C, no such association was found for advanced fibrosis in NAFLD patients (Table 1). However, unlike MRP2, BSEP protein levels were not reduced in animal models of NAFLD, although mRNA levels were also lower [125]. The role of BSEP in lipid metabolism was emphasized by a recent study revealing that cellular energy depletion activates AMPK–SRC-2 pathway which, via BSEP activation, promotes the intestinal absorption of dietary fat [126]. Therefore, pharmacologic therapies targeting BSEP and/or FXR may be beneficial in the management of NAFLD.

As another canalicular transporter, MRP2 may be involved in the pathogenesis of NAFLD. Downregulation of MRP2 in obese Zucker rats with defective leptin signaling [125] may lead to accumulation of potentially toxic metabolites. Furthermore, *MRP2* polymorphisms have been linked to NAFLD susceptibility and progression (Table 1). Interestingly, MRP2 expression was increased in *ob/ob* mice as another model of NAFLD and leptin deficiency [127].

Currently, no approved drugs for the treatment of NAFLD exist. UDCA is capable of modulating transporter expression [60,64,128], but clinical trials (including high dose studies) of NAFLD and NASH are rather disappointing [129]. In contrast to humans, taurine-conjugated UDCA reduces endoplasmic reticulum stress in mice thereby reversing insulin resistance [130] although this mechanism is still controversial [131]. Notably, high dose UDCA improves biochemical markers of liver injury, fibrosis and insulin resistance in patients with NASH [132].

The key role of FXR and TGR5 in regulation of lipid and glucose metabolism places these BA receptors in the center of interest for future management of NAFLD. In fact, several studies in animal models for obesity and NAFLD have recently demonstrated the beneficial effects of pharmacological stimulation of FXR and TGR5 on hepatic steatosis and insulin resistance. As such, the FXR agonist INT747 improves insulin sensitivity and liver function in humans [133], whereas another FXR agonist (WAY-362450) attenuates liver inflammation and fibrosis in a mouse model of fatty liver [134]. INT777, a TGR5 activator, reduces hepatic steatosis in mice, induced by high fat feeding, by promoting energy expenditure and GLP-1 secretion [37]. Taken together, a dual agonist activating both FXR and TGR5, such as the INT767, may be promising for NAFLD treatment.

GLP-1 receptor is also expressed in human hepatocytes [135] and reduced in NASH [136]. Moreover, GLP-1 treatment reduces steatosis in mice [137]. BA sequestrant-mediated activation of GLP-1 in the intestine (see above) could therefore also promote GLP-1 receptor activity in the liver that in turn increases PPARα and PPARγ as well as AMPK expression and stimulates β-oxidation, lipid storage and reactive oxygen species detoxification [136,137].

## Liver regeneration and cancer

Bile formation is impaired after partial hepatectomy (PH) [138] which results in cholestasis with elevated serum BA levels. Basolateral NTCP, OATP1 and OATP2 are downregulated [139,140], while the canalicular transporters MRP2 and BSEP are maintained or even increased after up to 70% PH [140,141]. However, loss of functional liver tissue by massive PH (up to 90%) is associated with diminished MRP2 expression and consecutive hyperbilirubinemia [142], which may contribute to postoperative jaundice. The molecular mechanisms involve cytokines such as IL6 and TNFα, key repressors of the basolateral transporter expression via HNF1α; conversely TNFα inactivation restores the transporter expression [143]. Intracellular BA overload after loss of functional liver mass can be counteracted by upregulation of the alternative basolateral BA export pumps MRP3 [142] and MRP4 [139]. The importance of these adaptive changes for liver regeneration is underlined by delayed liver regeneration in mice lacking *MRP3* with subsequently lowered BA concentration in the portal blood, and impaired FXR signaling [144].

Importantly, increased BA levels during liver injury may also have mechanistic relevance for liver repair. Interruption of the enterohepatic circulation delays liver regeneration [145] and BAs are able to stimulate hepatocyte proliferation [146], effects recently attributed to FXR and its downstream cell cycle regulator forkhead box m1b [147]. As such, elevated BAs accelerate liver growth and promote liver regeneration, which is in turn impaired in mice lacking FXR [148]. In addition to the protective role of hepatic FXR, intestinal FXR activation of FGF15 pathway protects the hepatocytes from cell death by repressing BA synthesis after PH [149,150]. In pregnancy, a condition known to be accompanied by gestational hepatomegaly, loss of FXR leads to impairment of liver growth [151].

Besides their role in promoting liver repair, BA exposure together with inflammatory stimuli may play a role in carcinogenesis. Mice lacking *FXR* or *Mdr2* with impaired BA homeostasis and inflammation spontaneously develop hepatocellular carcinoma (HCC) [152,153] and treatment with cholestyramine reduces tumor frequency [153]. Similar to human HCC, activation of the Wnt-β-catenin pathway is an early event even before tumor formation in *FXR* knockout mice [154]. Reduced FXR expression and activity have also been reported in human HCC [154] and in colon cancer [155]. Moreover, children with progressive familial intrahepatic cholestasis due to deficiency of the FXR target *BSEP* have an increased risk of HCC (Table 1) and cases of cholangiocarcinoma are described in patients with *BSEP* deficiency [156]. Reduced FXR expression has been recently shown in cholangiocellular carcinoma, while TGR5 has profoundly increased [157]. Preliminary data suggest that TGR5 may mediate resistance of cancer cells to apoptosis [157].

Treatment of HCC is limited by resistance to chemotherapy, which might be mediated by MDRs and MRPs and reduced hepatocellular drug uptake into HCCs, because of their capacity to export the anticancer drug out of expressing cells. The expression pattern of these transporters varies between individual patients [158]. Moreover, the expression of hepatobiliary (BA and organic anion) transporters is not only interesting for the therapeutic outcome, but also represents a determinant for radiological detection of HCC, since hepatocyte-selective enhancement with contrast agent on magnetic resonance images correlates with the expression pattern of OATPs and MRP2 [159]. Collectively, these findings underpin the diagnostic and therapeutic relevance of BA transporters for liver cancer.

## Summary and future directions

Hepatobiliary transporters are crucial to preserve bile formation and enterohepatic circulation of BAs. Within the enterohepatic circulation, BAs exert numerous functions such as facilitating intestinal lipid absorption, clearance of potentially toxic molecules and regulation of lipid, glucose and energy homeostasis. Disturbances in these delicate processes may result in a variety of hepatic, intestinal and systemic disorders. In addition, hepatic and intestinal diseases may induce secondary changes in transporter expression and regulation, thus affecting BA homeostasis. Understanding the mechanisms of BA transport and its regulation under (patho)physiologic conditions represents a powerful tool for the development of novel therapeutic approaches to many hepatic diseases with misbalanced BA metabolism. Targeting hepatobiliary transporters and their regulatory NRs should therefore open new therapeutic avenues for a broad range of diseases of the liver and beyond.

## Conflict of interest

M. Trauner has received research support from Intercept and Falk and is listed at the speaker’s bureaus of Gilead, Roche, Merck Sharp Dohme and Falk Foundation. The Medical University of Graz has filed a patent on the medical use of Nor-UDCA and M. Trauner is listed as co-inventor.

## Financial support

This work was supported by grants P 19118-B05, F3008-B05 and F3517 from the Austrian Science Foundation and European Community’s Seventh Framework Program (FP7/2007-2013) under grant agreement HEALTH-F2-2009-241762 for the project FLIP (to MT).

## Figures and Tables

**Table 1:** **Summary of hepatobiliary transporters in hepatocytes, their function, regulation through nuclear receptors and genetic alterations**. See supplementary material for all references in this table.

ABCG5/8, cholesterol efflux pump, ATP-binding cassette, subfamily G, member 5/8; BAs, bile acids; BCRP (ABCG2), breast cancer resistance protein, ATP-binding cassette, subfamily G, member 2; BRIC, benign recurrent intrahepatic cholestasis; BSEP (ABCB11), bile salt export pump; CA, cholic acid; CAR (NR1I3), constitutive androstane receptor; FXR (NR1H4), farnesoid X receptor/bile acid receptor; GR (NR3C1), glucocorticoid receptor; HCC, hepatocellular carcinoma; HNF4α (NR2A1), hepatocyte nuclear factor 4 alpha; IBD, inflammatory bowel disease, ICP, intrahepatic cholestasis of pregnancy; LXRα (NR1H3), liver X receptor alpha; MDR1 (ABCB1), *p*-glycoprotein, multidrug resistance protein 1, ATP-binding cassette, subfamily B, member 1; MDR2/3 (ABCB4), multidrug resistance protein 2/3; MRP2 (ABCC2), multidrug resistanceassociated protein 2, ATP-binding cassette, subfamily C, member 2; MRP3 (ABCC3) multidrug resistance-associated protein 3, ATP-binding cassette, subfamily C, member 3; MRP4 (ABCC4) multidrug resistance-associated protein 4, ATP-binding cassette, subfamily C, member 4; NAFLD, non-alcoholic fatty liver disease; NTCP (SLC10A1), sodium/taurocholate cotransporting polypeptide, solute carrier family 10, member 1; OATP1A2 (SLCO1A2, OATP1, OATP-A, SLC21A3), solute carrier organic anion transporter family, member 1A2; OATP1B1 (SLCO1B1, OATP2, OATP-C, SLC21A6), solute carrier organic anion transporter family, member 1B1; OATP1B3 (SLCO1B3, OATP8, SLC21A8) solute carrier organic anion transporter family, member 1B3; OSTα/β, organic solute transporter alpha/beta; PBC, primary biliary cirrhosis; PFIC, progressive familial intrahepatic cholestasis; PPARα (NR1C1), peroxisome proliferator-activated receptor alpha; PSC, primary sclerosing cholangitis; PXR (NR1I2), pregnane X receptor; RXRα (NR2B1), retinoid X receptor alpha; SHP (NR0B2), short heterodimer partner; VDR (NR1I1), vitamin D receptor.

## Appendix A Supplementary data

Supplementary References
