# Supplementary material for: Bile acid transporters and regulatory nuclear receptors in the liver and beyond
Source: J Hepatol. 2013 Jan;58(1):155–68. doi: 10.1016/j.jhep.2012.08.002 (PMC3526785; doi:10.1016/j.jhep.2012.08.002)
Supplement: Supplementary References [file mmc1.docx]

**Supplementary References**

[1] Trauner M, Boyer JL. Bile salt transporters: molecular characterization, function, and regulation. Physiol Rev 2003;83:633-671.

[2] Keppler D. Multidrug resistance proteins (MRPs, ABCCs): importance for pathophysiology and drug therapy. Handb Exp Pharmacol 2011:299-323.

[3] Hagenbuch B, Gui C. Xenobiotic transporters of the human organic anion transporting polypeptides (OATP) family. Xenobiotica 2008;38:778-801.

[4] Stieger B. The role of the sodium-taurocholate cotransporting polypeptide (NTCP) and of the bile salt export pump (BSEP) in physiology and pathophysiology of bile formation. Handb Exp Pharmacol 2011:205-259.

[5] Ballatori N. Pleiotropic functions of the organic solute transporter Ostalpha-Ostbeta. Dig Dis 2011;29:13-17.

[6] Wagner M, Zollner G, Trauner M. Nuclear receptor regulation of the adaptive response of bile acid transporters in cholestasis. Semin Liver Dis 2010;30:160-177.

[7] Geier A, Wagner M, Dietrich CG, Trauner M. Principles of hepatic organic anion transporter regulation during cholestasis, inflammation and liver regeneration. Biochim Biophys Acta 2007;1773:283-308.

[8] Pan W, Song IS, Shin HJ, Kim MH, Choi YL, Lim SJ, et al. Genetic polymorphisms in Na+-taurocholate co-transporting polypeptide (NTCP) and ileal apical sodium-dependent bile acid transporter (ASBT) and ethnic comparisons of functional variants of NTCP among Asian populations. Xenobiotica 2011;41:501-510.

[9] Choi MK, Shin HJ, Choi YL, Deng JW, Shin JG, Song IS. Differential effect of genetic variants of Na(+)-taurocholate co-transporting polypeptide (NTCP) and organic anion-transporting polypeptide 1B1 (OATP1B1) on the uptake of HMG-CoA reductase inhibitors. Xenobiotica 2011;41:24-34.

[10] Gong L, Aranibar N, Han YH, Zhang Y, Lecureux L, Bhaskaran V, et al. Characterization of organic anion-transporting polypeptide (Oatp) 1a1 and 1a4 null mice reveals altered transport function and urinary metabolomic profiles. Toxicol Sci 2011;122:587-597.

[11] Lee W, Glaeser H, Smith LH, Roberts RL, Moeckel GW, Gervasini G, et al. Polymorphisms in human organic anion-transporting polypeptide 1A2 (OATP1A2): implications for altered drug disposition and central nervous system drug entry. J Biol Chem 2005;280:9610-9617.

[12] Yamakawa Y, Hamada A, Shuto T, Yuki M, Uchida T, Kai H, et al. Pharmacokinetic impact of SLCO1A2 polymorphisms on imatinib disposition in patients with chronic myeloid leukemia. Clin Pharmacol Ther 2011;90:157-163.

[13] Franke RM, Scherkenbach LA, Sparreboom A. Pharmacogenetics of the organic anion transporting polypeptide 1A2. Pharmacogenomics 2009;10:339-344.

[14] Csanaky IL, Lu H, Zhang Y, Ogura K, Choudhuri S, Klaassen CD. Organic anion-transporting polypeptide 1b2 (Oatp1b2) is important for the hepatic uptake of unconjugated bile acids: Studies in Oatp1b2-null mice. Hepatology 2011;53:272-281.

[15] Lu H, Choudhuri S, Ogura K, Csanaky IL, Lei X, Cheng X, et al. Characterization of organic anion transporting polypeptide 1b2-null mice: essential role in hepatic uptake/toxicity of phalloidin and microcystin-LR. Toxicological sciences : an official journal of the Society of Toxicology 2008;103:35-45.

[16] Meyer zu Schwabedissen HE, Ware JA, Finkelstein D, Chaudhry AS, Mansell S, Leon-Ponte M, et al. Hepatic organic anion transporting polypeptide transporter and thyroid hormone receptor interplay determines cholesterol and glucose homeostasis. Hepatology 2011;54:644-654.

[17] Johnson AD, Kavousi M, Smith AV, Chen MH, Dehghan A, Aspelund T, et al. Genome-wide association meta-analysis for total serum bilirubin levels. Hum Mol Genet 2009;18:2700-2710.

[18] Buch S, Schafmayer C, Volzke H, Seeger M, Miquel JF, Sookoian SC, et al. Loci from a genome-wide analysis of bilirubin levels are associated with gallstone risk and composition. Gastroenterology 2010;139:1942-1951 e1942.

[19] Miura M, Kagaya H, Satoh S, Inoue K, Saito M, Habuchi T, et al. Influence of drug transporters and UGT polymorphisms on pharmacokinetics of phenolic glucuronide metabolite of mycophenolic acid in Japanese renal transplant recipients. Ther Drug Monit 2008;30:559-564.

[20] Lam P, Wang R, Ling V. Bile acid transport in sister of P-glycoprotein (ABCB11) knockout mice. Biochemistry 2005;44:12598-11605.

[21] Wang R, Salem M, Yousef IM, Tuchweber B, Lam P, Childs SJ, et al. Targeted inactivation of sister of P-glycoprotein gene (spgp) in mice results in nonprogressive but persistent intrahepatic cholestasis. Proc Natl Acad Sci U S A 2001;98:2011-2016.

[22] Wang R, Chen HL, Liu L, Sheps JA, Phillips MJ, Ling V. Compensatory role of P-glycoproteins in knockout mice lacking the bile salt export pump. Hepatology 2009;50:948-956.

[23] Wang R, Lam P, Liu L, Forrest D, Yousef IM, Mignault D, et al. Severe cholestasis induced by cholic acid feeding in knockout mice of sister of P-glycoprotein. Hepatology 2003;38:1489-1499.

[24] Strautnieks SS, Bull LN, Knisely AS, Kocoshis SA, Dahl N, Arnell H, et al. A gene encoding a liver-specific ABC transporter is mutated in progressive familial intrahepatic cholestasis. Nat Genet 1998;20:233-238.

[25] Strautnieks SS, Byrne JA, Pawlikowska L, Cebecauerova D, Rayner A, Dutton L, et al. Severe bile salt export pump deficiency: 82 different ABCB11 mutations in 109 families. Gastroenterology 2008;134:1203-1214.

[26] Knisely AS, Strautnieks SS, Meier Y, Stieger B, Byrne JA, Portmann BC, et al. Hepatocellular carcinoma in ten children under five years of age with bile salt export pump deficiency. Hepatology 2006;44:478-486.

[27] van Mil SW, van der Woerd WL, van der Brugge G, Sturm E, Jansen PL, Bull LN, et al. Benign recurrent intrahepatic cholestasis type 2 is caused by mutations in ABCB11. Gastroenterology 2004;127:379-384.

[28] Dixon PH, van Mil S, Chambers J, Strautnieks S, Thompson R, Lammert F, et al. Contribution of Variant Alleles of ABCB11 to Susceptibility to Intrahepatic Cholestasis of Pregnancy. Gut 2008;58:537-544.

[29] Eloranta ML, Hakli T, Hiltunen M, Helisalmi S, Punnonen K, Heinonen S. Association of single nucleotide polymorphisms of the bile salt export pump gene with intrahepatic cholestasis of pregnancy. Scand J Gastroenterol 2003;38:648-652.

[30] Meier Y, Zodan T, Lang C, Zimmermann R, Kullak-Ublick GA, Meier PJ, et al. Increased susceptibility for intrahepatic cholestasis of pregnancy and contraceptive-induced cholestasis in carriers of the 1331T>C polymorphism in the bile salt export pump. World J Gastroenterol 2008;14:38-45.

[31] Iwata R, Baur K, Stieger B, Mertens JC, Daly AK, Frei P, et al. A common polymorphism in the ABCB11 gene is associated with advanced fibrosis in hepatitis C but not in non-alcoholic fatty liver disease. Clin Sci (Lond) 2011;120:287-296.

[32] Iwata R, Stieger B, Mertens JC, Muller T, Baur K, Frei P, et al. The role of bile acid retention and a common polymorphism in the ABCB11 gene as host factors affecting antiviral treatment response in chronic hepatitis C. J Viral Hepat 2010.

[33] Lang C, Meier Y, Stieger B, Beuers U, Lang T, Kerb R, et al. Mutations and polymorphisms in the bile salt export pump and the multidrug resistance protein 3 associated with drug-induced liver injury. Pharmacogenet Genomics 2007;17:47-60.

[34] Buchler M, Konig J, Brom M, Kartenbeck J, Spring H, Horie T, et al. cDNA cloning of the hepatocyte canalicular isoform of the multidrug resistance protein, cMrp, reveals a novel conjugate export pump deficient in hyperbilirubinemic mutant rats. J Biol Chem 1996;271:15091-15098.

[35] Paulusma CC, Bosma PJ, Zaman GJ, Bakker CT, Otter M, Scheffer GL, et al. Congenital jaundice in rats with a mutation in a multidrug resistance-associated protein gene. Science 1996;271:1126-1128.

[36] Chu XY, Strauss JR, Mariano MA, Li J, Newton DJ, Cai X, et al. Characterization of mice lacking the multidrug resistance protein MRP2 (ABCC2). J Pharmacol Exp Ther 2006;317:579-589.

[37] Vlaming ML, Mohrmann K, Wagenaar E, de Waart DR, Elferink RP, Lagas JS, et al. Carcinogen and anticancer drug transport by Mrp2 in vivo: studies using Mrp2 (Abcc2) knockout mice. J Pharmacol Exp Ther 2006;318:319-327.

[38] Paulusma CC, Kool M, Bosma PJ, Scheffer GL, ter Borg F, Scheper RJ, et al. A mutation in the human canalicular multispecific organic anion transporter gene causes the Dubin-Johnson syndrome. Hepatology 1997;25:1539-1542.

[39] Sookoian S, Castano G, Gianotti TF, Gemma C, Pirola CJ. Polymorphisms of MRP2 (ABCC2) are associated with susceptibility to nonalcoholic fatty liver disease. J Nutr Biochem 2009;20:765-770.

[40] Daly AK, Aithal GP, Leathart JB, Swainsbury RA, Dang TS, Day CP. Genetic susceptibility to diclofenac-induced hepatotoxicity: contribution of UGT2B7, CYP2C8, and ABCC2 genotypes. Gastroenterology 2007;132:272-281.

[41] Hoblinger A, Grunhage F, Sauerbruch T, Lammert F. Association of the c.3972C>T variant of the multidrug resistance-associated protein 2 Gene (MRP2/ABCC2) with susceptibility to bile duct cancer. Digestion 2009;80:36-39.

[42] Foucaud-Vignault M, Soayfane Z, Menez C, Bertrand-Michel J, Martin PG, Guillou H, et al. P-glycoprotein Dysfunction Contributes to Hepatic Steatosis and Obesity in Mice. PLoS One 2011;6:e23614.

[43] Schwab M, Schaeffeler E, Marx C, Fromm MF, Kaskas B, Metzler J, et al. Association between the C3435T MDR1 gene polymorphism and susceptibility for ulcerative colitis. Gastroenterology 2003;124:26-33.

[44] Andersen V, Ostergaard M, Christensen J, Overvad K, Tjonneland A, Vogel U. Polymorphisms in the xenobiotic transporter Multidrug Resistance 1 (MDR1) and interaction with meat intake in relation to risk of colorectal cancer in a Danish prospective case-cohort study. BMC Cancer 2009;9:407.

[45] Mennone A, Soroka CJ, Harry KM, Boyer JL. Role of breast cancer resistance protein in the adaptive response to cholestasis. Drug Metab Dispos 2010;38:1673-1678.

[46] Blazquez AM, Briz O, Romero MR, Rosales R, Monte MJ, Vaquero J, et al. Characterization of the Role of ABCG2 as a Bile Acid Transporter in Liver and Placenta. Mol Pharmacol 2011.

[47] Imanishi H, Okamura N, Yagi M, Noro Y, Moriya Y, Nakamura T, et al. Genetic polymorphisms associated with adverse events and elimination of methotrexate in childhood acute lymphoblastic leukemia and malignant lymphoma. J Hum Genet 2007;52:166-171.

[48] Yanagimachi M, Naruto T, Hara T, Kikuchi M, Hara R, Miyamae T, et al. Influence of polymorphisms within the methotrexate pathway genes on the toxicity and efficacy of methotrexate in patients with juvenile idiopathic arthritis. Br J Clin Pharmacol 2011;71:237-243.

[49] Bailey KM, Romaine SP, Jackson BM, Farrin AJ, Efthymiou M, Barth JH, et al. Hepatic metabolism and transporter gene variants enhance response to rosuvastatin in patients with acute myocardial infarction: the GEOSTAT-1 Study. Circ Cardiovasc Genet 2010;3:276-285.

[50] Urquhart BL, Ware JA, Tirona RG, Ho RH, Leake BF, Schwarz UI, et al. Breast cancer resistance protein (ABCG2) and drug disposition: intestinal expression, polymorphisms and sulfasalazine as an in vivo probe. Pharmacogenet Genomics 2008;18:439-448.

[51] Smit JJ, Schinkel AH, Oude Elferink RP, Groen AK, Wagenaar E, van Deemter L, et al. Homozygous disruption of the murine mdr2 P-glycoprotein gene leads to a complete absence of phospholipid from bile and to liver disease. Cell 1993;75:451-462.

[52] Fickert P, Fuchsbichler A, Wagner M, Zollner G, Kaser A, Tilg H, et al. Regurgitation of bile acids from leaky bile ducts causes sclerosing cholangitis in Mdr2 (Abcb4) knockout mice. Gastroenterology 2004;127:261-274.

[53] Fickert P, Zollner G, Fuchsbichler A, Stumptner C, Weiglein AH, Lammert F, et al. Ursodeoxycholic acid aggravates bile infarcts in bile duct-ligated and Mdr2 knockout mice via disruption of cholangioles. Gastroenterology 2002;123:1238-1251.

[54] Lammert F, Wang DQ, Hillebrandt S, Geier A, Fickert P, Trauner M, et al. Spontaneous cholecysto- and hepatolithiasis in Mdr2-/- mice: a model for low phospholipid-associated cholelithiasis. Hepatology 2004;39:117-128.

[55] Mauad TH, van Nieuwkerk CM, Dingemans KP, Smit JJ, Schinkel AH, Notenboom RG, et al. Mice with homozygous disruption of the mdr2 P-glycoprotein gene. A novel animal model for studies of nonsuppurative inflammatory cholangitis and hepatocarcinogenesis. Am J Pathol 1994;145:1237-1245.

[56] Katzenellenbogen M, Pappo O, Barash H, Klopstock N, Mizrahi L, Olam D, et al. Multiple adaptive mechanisms to chronic liver disease revealed at early stages of liver carcinogenesis in the Mdr2-knockout mice. Cancer Res 2006;66:4001-4010.

[57] Pikarsky E, Porat RM, Stein I, Abramovitch R, Amit S, Kasem S, et al. NF-kappaB functions as a tumour promoter in inflammation-associated cancer. Nature 2004;431:461-466.

[58] de Vree JM, Jacquemin E, Sturm E, Cresteil D, Bosma PJ, Aten J, et al. Mutations in the MDR3 gene cause progressive familial intrahepatic cholestasis. Proc Natl Acad Sci U S A 1998;95:282-287.

[59] Deleuze JF, Jacquemin E, Dubuisson C, Cresteil D, Dumont M, Erlinger S, et al. Defect of multidrug-resistance 3 gene expression in a subtype of progressive familial intrahepatic cholestasis. Hepatology 1996;23:904-908.

[60] Pauli-Magnus C, Lang T, Meier Y, Zodan-Marin T, Jung D, Breymann C, et al. Sequence analysis of bile salt export pump (ABCB11) and multidrug resistance p-glycoprotein 3 (ABCB4, MDR3) in patients with intrahepatic cholestasis of pregnancy. Pharmacogenetics 2004;14:91-102.

[61] Schneider G, Paus TC, Kullak-Ublick GA, Meier PJ, Wienker TF, Lang T, et al. Linkage between a new splicing site mutation in the MDR3 alias ABCB4 gene and intrahepatic cholestasis of pregnancy. Hepatology 2007;45:150-158.

[62] Floreani A, Carderi I, Paternoster D, Soardo G, Azzaroli F, Esposito W, et al. Hepatobiliary phospholipid transporter ABCB4, MDR3 gene variants in a large cohort of Italian women with intrahepatic cholestasis of pregnancy. Dig Liver Dis 2008;40:366-370.

[63] Ziol M, Barbu V, Rosmorduc O, Frassati-Biaggi A, Barget N, Hermelin B, et al. ABCB4 heterozygous gene mutations associated with fibrosing cholestatic liver disease in adults. Gastroenterology 2008;135:131-141.

[64] Tomaiuolo R, Degiorgio D, Coviello DA, Baccarelli A, Elce A, Raia V, et al. An MBL2 haplotype and ABCB4 variants modulate the risk of liver disease in cystic fibrosis patients: a multicentre study. Dig Liver Dis 2009;41:817-822.

[65] Ohishi Y, Nakamura M, Iio N, Higa S, Inayoshi M, Aiba Y, et al. Single-nucleotide polymorphism analysis of the multidrug resistance protein 3 gene for the detection of clinical progression in Japanese patients with primary biliary cirrhosis. Hepatology 2008;48:853-862.

[66] Pauli-Magnus C, Kerb R, Fattinger K, Lang T, Anwald B, Kullak-Ublick GA, et al. BSEP and MDR3 haplotype structure in healthy Caucasians, primary biliary cirrhosis and primary sclerosing cholangitis. Hepatology 2004;39:779-791.

[67] Rosmorduc O, Hermelin B, Boelle PY, Parc R, Taboury J, Poupon R. ABCB4 gene mutation-associated cholelithiasis in adults. Gastroenterology 2003;125:452-459.

[68] Rosmorduc O, Hermelin B, Poupon R. MDR3 gene defect in adults with symptomatic intrahepatic and gallbladder cholesterol cholelithiasis. Gastroenterology 2001;120:1459-1467.

[69] Rosmorduc O, Poupon R. Low phospholipid associated cholelithiasis: association with mutation in the MDR3/ABCB4 gene. Orphanet J Rare Dis 2007;2:29.

[70] Plosch T, Bloks VW, Terasawa Y, Berdy S, Siegler K, Van Der Sluijs F, et al. Sitosterolemia in ABC-transporter G5-deficient mice is aggravated on activation of the liver-X receptor. Gastroenterology 2004;126:290-300.

[71] Klett EL, Lu K, Kosters A, Vink E, Lee MH, Altenburg M, et al. A mouse model of sitosterolemia: absence of Abcg8/sterolin-2 results in failure to secrete biliary cholesterol. BMC Med 2004;2:5.

[72] Wang HH, Patel SB, Carey MC, Wang DQ. Quantifying anomalous intestinal sterol uptake, lymphatic transport, and biliary secretion in Abcg8(-/-) mice. Hepatology 2007;45:998-1006.

[73] Yu L, Hammer RE, Li-Hawkins J, Von Bergmann K, Lutjohann D, Cohen JC, et al. Disruption of Abcg5 and Abcg8 in mice reveals their crucial role in biliary cholesterol secretion. Proc Natl Acad Sci U S A 2002;99:16237-16242.

[74] Lee MH, Lu K, Hazard S, Yu H, Shulenin S, Hidaka H, et al. Identification of a gene, ABCG5, important in the regulation of dietary cholesterol absorption. Nat Genet 2001;27:79-83.

[75] Berge KE, Tian H, Graf GA, Yu L, Grishin NV, Schultz J, et al. Accumulation of dietary cholesterol in sitosterolemia caused by mutations in adjacent ABC transporters. Science 2000;290:1771-1775.

[76] Katsika D, Magnusson P, Krawczyk M, Grunhage F, Lichtenstein P, Einarsson C, et al. Gallstone disease in Swedish twins: risk is associated with ABCG8 D19H genotype. J Intern Med 2010;268:279-285.

[77] Grunhage F, Acalovschi M, Tirziu S, Walier M, Wienker TF, Ciocan A, et al. Increased gallstone risk in humans conferred by common variant of hepatic ATP-binding cassette transporter for cholesterol. Hepatology 2007;46:793-801.

[78] Wang Y, Jiang ZY, Fei J, Xin L, Cai Q, Jiang ZH, et al. ATP binding cassette G8 T400K polymorphism may affect the risk of gallstone disease among Chinese males. Clin Chim Acta 2007;384:80-85.

[79] Acalovschi M, Ciocan A, Mostean O, Tirziu S, Chiorean E, Keppeler H, et al. Are plasma lipid levels related to ABCG5/ABCG8 polymorphisms? A preliminary study in siblings with gallstones. Eur J Intern Med 2006;17:490-494.

[80] Buch S, Schafmayer C, Volzke H, Becker C, Franke A, von Eller-Eberstein H, et al. A genome-wide association scan identifies the hepatic cholesterol transporter ABCG8 as a susceptibility factor for human gallstone disease. Nat Genet 2007;39:995-999.

[81] Kajinami K, Brousseau ME, Nartsupha C, Ordovas JM, Schaefer EJ. ATP binding cassette transporter G5 and G8 genotypes and plasma lipoprotein levels before and after treatment with atorvastatin. J Lipid Res 2004;45:653-656.

[82] Stender S, Frikke-Schmidt R, Nordestgaard BG, Tybjaerg-Hansen A. Sterol transporter adenosine triphosphate-binding cassette transporter G8, gallstones, and biliary cancer in 62,000 individuals from the general population. Hepatology 2010.

[83] Xu HL, Cheng JR, Andreotti G, Gao YT, Rashid A, Wang BS, et al. Cholesterol metabolism gene polymorphisms and the risk of biliary tract cancers and stones: a population-based case-control study in Shanghai, China. Carcinogenesis 2011;32:58-62.

[84] Belinsky MG, Dawson PA, Shchaveleva I, Bain LJ, Wang R, Ling V, et al. Analysis of the in vivo functions of Mrp3. Mol Pharmacol 2005;68:160-168.

[85] Manautou JE, de Waart DR, Kunne C, Zelcer N, Goedken M, Borst P, et al. Altered disposition of acetaminophen in mice with a disruption of the Mrp3 gene. Hepatology 2005;42:1091-1098.

[86] Lang T, Hitzl M, Burk O, Mornhinweg E, Keil A, Kerb R, et al. Genetic polymorphisms in the multidrug resistance-associated protein 3 (ABCC3, MRP3) gene and relationship to its mRNA and protein expression in human liver. Pharmacogenetics 2004;14:155-164.

[87] Mennone A, Soroka CJ, Cai SY, Harry K, Adachi M, Hagey L, et al. Mrp4-/- mice have an impaired cytoprotective response in obstructive cholestasis. Hepatology 2006;43:1013-1021.

[88] Ban H, Andoh A, Imaeda H, Kobori A, Bamba S, Tsujikawa T, et al. The multidrug-resistance protein 4 polymorphism is a new factor accounting for thiopurine sensitivity in Japanese patients with inflammatory bowel disease. J Gastroenterol 2010;45:1014-1021.

[89] Soroka CJ, Mennone A, Hagey LR, Ballatori N, Boyer JL. Mouse organic solute transporter alpha deficiency enhances renal excretion of bile acids and attenuates cholestasis. Hepatology 2009.

[90] Ballatori N, Fang F, Christian WV, Li N, Hammond CL. Ostalpha-Ostbeta is required for bile acid and conjugated steroid disposition in the intestine, kidney, and liver. Am J Physiol Gastrointest Liver Physiol 2008;295:G179-G186.

[91] Rao A, Haywood J, Craddock AL, Belinsky MG, Kruh GD, Dawson PA. The organic solute transporter {alpha}-{beta}, Ost{alpha}-Ost{beta}, is essential for intestinal bile acid transport and homeostasis. Proc Natl Acad Sci U S A 2008.

[92] Renner O, Harsch S, Strohmeyer A, Schimmel S, Stange EF. Reduced ileal expression of OSTalpha-OSTbeta in non-obese gallstone disease. Journal of lipid research 2008;49:2045-2054.
